# Effect of Traditional Chinese Medicine on Treating Antibiotic-Associated Diarrhea in Children: A Systematic Review and Meta-Analysis

**DOI:** 10.1155/2022/6108772

**Published:** 2022-09-22

**Authors:** Shunlian Fu, Lisha Sun, Yiding Chen, Qian Zhou, Lijun Yuan, Zinan Li, Qiu Chen

**Affiliations:** Hospital of Chengdu University of Traditional Chinese Medicine, chengdu, China

## Abstract

**Background:**

Due to the limited treatment options in antibiotic-associated diarrhea (AAD) in children, more effective treatments should be explored. Traditional Chinese medicine (TCM) has a long history in China, which has produced a pretty effect in clinical practice. Many randomized clinical trials (RCTs) have explored the effect of traditional Chinese medicine on treating AAD in children. However, there has been no systematic review or meta-analysis on the impact of TCM on AAD in children. The aim of this study was to systematically review RCTs on the effect of TCM in children with AAD.

**Methods:**

RCTs in the past ten years on TCM for AAD in children were included. We searched Electronic databases as much as possible. This paper was registered in PROSPERO (CRD42022301034).

**Results:**

26 studies were included in this systematic review. 25 studies reported the effects of TCM interventions on the total effective rate (RR = 1.20, CI 1.16 to 1.24; *p* < 0.001). 7 studies reported the effects of TCM interventions on the time to change the shape of feces (MD = −1.37, CI −1.67 to −1.07; *p* < 0.001). 17 studies reported the effects of TCM interventions (MD = −1.43, CI −1.71 to −1.15; *p* < 0.001). The pooled results showed that there were no significant differences between the two groups in CD3+, CD4+, CD8+, CD4 : CD8, time for bowel sounds to return to normal, hs-CRP, and IgM. There was a significant difference between the two groups in frequency of diarrhea on the third day after TCM intervention, vomiting improvement time, diamine oxidase, IL-8, TNF, IgA, IgG, and average hospital stay.

**Conclusions:**

TCM interventions combined with conventional therapy can improve the therapeutic effect of AAD in children. However, future studies are still needed for the low methodological quality.

## 1. Introduction

As one of the most widely used drugs in subjects between birth and 18 years of age, oral or intravenous antibiotics are applied to more than half of those subjects, resulting in many adverse events, such as antibiotic-associated diarrhea (AAD) [1, 2]. AAD is a common complication in up to a third of all patients treated with antibiotics, and in particular in 11–62% of children and in up to 80% of hospitalized toddlers [3–5]. AAD may result in withdrawing antibiotics, rehydrating by vein, or receiving treatment in hospital, complexing the treatment of underlying infection [6]. At present, about the treatment of AAD in children, suggestions are recommended as follows: discontinuation and adjustment of antibiotics, probiotics, treatment of specific pathogens, fecal microbiota transplantation, symptomatic and supportive treatment, surgical operation, and control of infection in hospital [7]. However, there are some limitations to the present treatments mentioned above. Opportunistic infections and translocations can be caused through the gastrointestinal tract when using probiotic bacteria, which may further become the causative agent of many severe human infections, including bacteremia, meningitis, and endocarditis [1]. Discontinuation and modulation of antibiotics may also aggravate the primary disease. Due to the immature technology of fecal microbiota transplantation, promotions are limited [8]. In addition, individual variability can always make an influence the therapeutic effect for patients [9]. Therefore, more effective treatments and alternative therapies need to be forced and explored.

As a primary complementary and alternative medicine, traditional Chinese medicine (TCM) has been widely used for thousands of years in China, which also has been accepted by more and more countries. Based on unique theories accumulated through phenotypic and individualized evidence in long-term clinical trials, TCM has been confirmed to play a crucial part in diarrhea and evidence also has been demonstrated from some systematic reviews and meta-analyses in recent years [10–14]. Recently, a series of randomized clinical trials (RCTs) have explored the effect of traditional Chinese medicine on treating AAD in children. However, no systematic review or meta-analysis has been conducted on the impact of TCM on AAD in children so far. The aim of this study was to systematically and comprehensively summarize RCTs of TCM interventions involving patients with AAD in children and to evaluate the efficacy of TCM in improving AAD.

## 2. Materials and Methods

We conducted this systematic review by reference to the Preferred Reporting Items for Systematic Reviews and Meta-Analysis (PRISMA) statement [15]. This paper were registered in PROSPERO (CRD42022301034).

### 2.1. Eligibility Criteria

We checked each article by following inclusion criteria as follows: (1) participants: the participants were patients with AAD in children; (2) interventions: on the basis of the control treatment, TCM interventions were given by oral administration in the experimental group; the interventions in the experimental group included any structured and conceptualized traditional Chinese medicine (TCM), such as Chinese herbal compounds, Chinese patent medicine, and single Chinese medical herbs, administered in the form of decoctions, granules, or powders [9, 16]. We did not restrict the dose, frequency, or duration; (3) comparison: the comparison was set as conventional western medicine treatment; (4) outcome: the primary outcomes were the total effective rate; the secondary outcomes were indicators associated with improvement of diarrhea symptoms, such as the time to change the shape of feces, antidiarrheal time, immune-related indicators, inflammatory biomarkers, average hospital stay, and diamine oxidase. All RCTs were published in the past ten years. The diagnosis of AAD is diarrhea after being exposed to antibiotics, whether on antibiotics or after discontinuing antibiotics for two months or longer [4, 5]. Etiologies for pediatric AAD may be in connection with viruses or *C. difficile*, but may also be related to osmotic imbalances in the intestines after antibiotic exposure and microbiota disruption [17].

The exclusion criteria were as follows: studies were excluded when the interventions in the experimental group were herb extracts, or any nondrug therapy combined with TCM, such as Chinese herb point application therapy, traditional Chinese medical massage, Chinese medicine rectal drops, and moxibustion. The control group included individuals treated with either Western medicine or placebo or treated with no intervention. If the intervention in the experimental group was based on Western medicine, the use of Western medicine in the control group should be consistent. Studies were excluded when the purpose of the study was to investigate the prevention of antibiotic-associated diarrhea by TCM. Articles about conferences, abstracts, and correspondence were excluded. Articles without complete data after consulting corresponding authors were excluded.

### 2.2. Search Strategy

Electronic databases were searched as follows: Web of Science, PubMed, EMBASE, Cochrane Library, China National Knowledge Infrastructure (CNKI), Wanfang Database, and VIP Information-Chinese Scientific Journal Database from inception to February 24th, 2022 in the English and Chinese languages. Key terms were “traditional Chinese medicine,” “randomized controlled trial,” “effective rate,” and “antibiotic-associated diarrhea.” Search terms are shown in Table 1. Grey literature were also included. When necessary, for collecting missing data on the main endpoints or consulting unclear information, the corresponding authors of the included systematic reviews were contacted.

### 2.3. Selection of Studies

All the search results based on the predefined search terms were imported into Endnote X9 (a reference management software). Two authors manually and independently screened the relevant literature by reading titles and abstracts, and then evaluated the full text on the basis of the eligibility criteria using a study-selection form. Any disagreements were solved by consulting a third researcher.

### 2.4. Quality Assessment

Two reviewers independently assessed the quality of all included studies by using the Cochrane Collaborations tool [18]. Criteria were as follows: random sequence generation, allocation concealment, blinding of outcome assessment or participant, incomplete outcome data, selective reporting, and other bias. The evaluation of each item was classified as low, high, or unclear risk according to the descriptions of the method in each study. Any disagreement was settled by discussing with the third author.

### 2.5. Data Extraction

Two authors independently extracted the data as follows: name of researcher, the included population, published year, the number of people included in the intervention group and in the control group, characteristics of participants, study types, intervention and dosage of TCM, treatment course, control group, baseline, treatment outcomes, adverse events, and other information. A third author checked the data extracted again. Disagreements were settled by discussing with the third author. When necessary, additional information were contacted with the corresponding authors of the studies. Two authors independently assessed the methodological quality and risk of bias (ROB) of all included studies by using the Cochrane Collaboration's tool.

### 2.6. Data Synthesis and Statistical Analyses

When the reported data for included outcomes were sufficient, we performed meta-analysis. Meta-analysis was conducted for the primary outcomes and the secondary outcomes,which were managed by using Review Manager (version 5.4; Cochrane Collaboration). In addition to total effective rate, risk ratio (RR) was used as the pooled effect size, and other outcome measures used mean difference as the pooled effect size(95% CI). When *p* values were less than 0.05, the results were considered statistically significant. Heterogeneity was evaluated by using I^2^ statistics: if I^2^ < 50%, a fixed-effect (FE) model was used; if I^2^ ≥ 50%, the random-effect (RE) model was used. The Chi-square test (*p* < 0.1) was also used to evaluate the heterogeneity. When there was heterogeneity, subgroup analysis was conducted. Exploration of publication bias was conducted when the included trials were more than ten in outcomes.

## 3. Results

**Figure 1:**
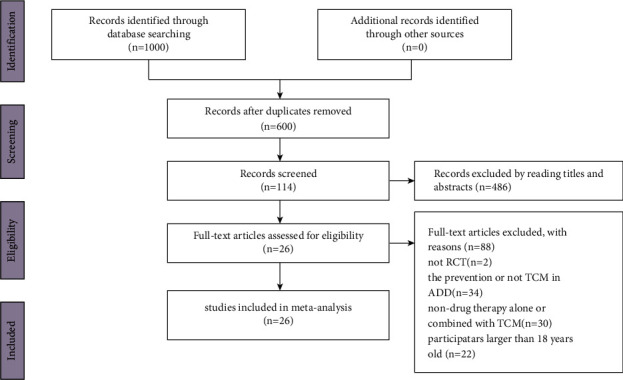
PRISMA flow diagram of the study-selection process.

In all, 1000 potentially relevant studies were yielded through the initial target databases search. After removing duplicates and eliminating ineligible studies, 114 articles were remained. After the full-text review, 88 studies were excluded, whose contents were irrelevant with our outcomes. The study selection process is shown in Figure 1. At last, 26 RCTs [19–44] were included in this systematic review.

### 3.1. Characteristics of the Trials Included in the Study

The total number of participants across the included studies was 2636, and the sample size in each study ranged from 50 to 165 participants. All included studies were published in China and the ethnicity of the participants were not reported. Treatment course ranged from 3 days to 2 weeks. In the included studies, 22 articles were reported about Chinese patent medicine, including Shenling Baizhu granule, Xingpi Yanger granule, Erxieting granules, Weichangan pill, modified Yigong Powder, Xiaoer Fuxie powder, Yiersan, Guben Yichang tablets, Buzhong Yiqi particles, and Qiweibaizhu powder. Details are shown in Table 2.

## 4. Methodological Quality

**Figure 2:**
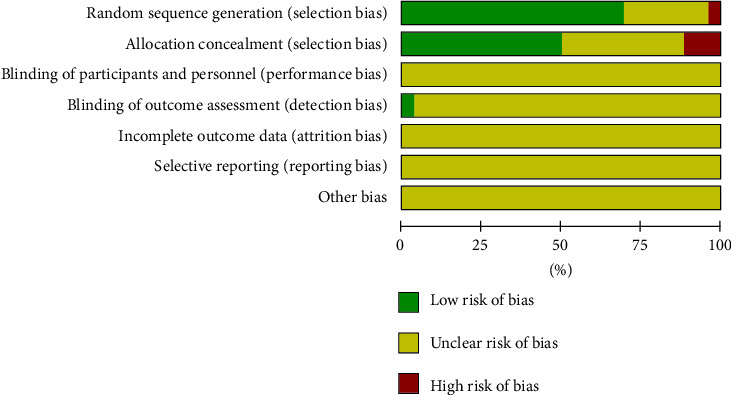
Risk of bias graph.

**Figure 3:**
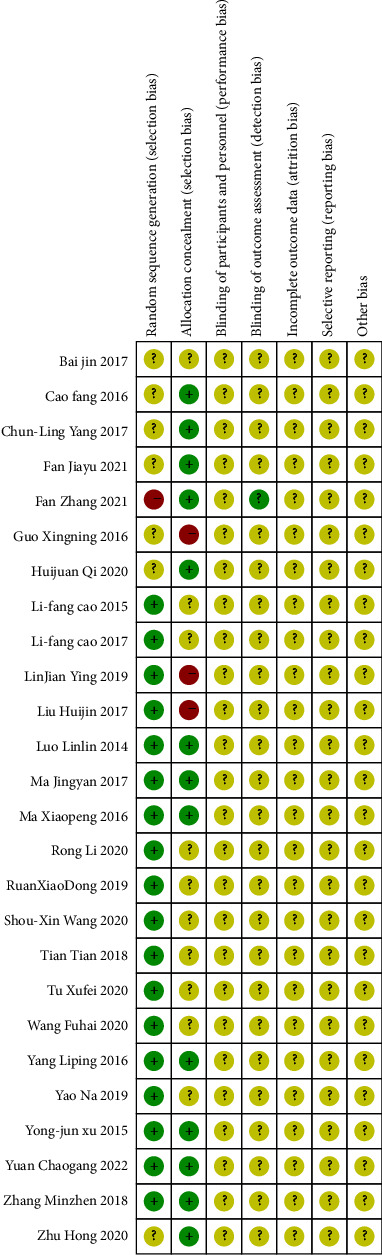
Risk of bias summary.

According to the results of Cochrane Collaboration risk of bias tool, the method of randomization and allocation concealment was described clearly in all included studies. The bias for each trial is shown in Figure 2, and the bias summary is shown in Figure 3.

### 4.1. Effect of TCM Combined with Conventional Therapy on the Total Effective Rate

**Figure 4:**
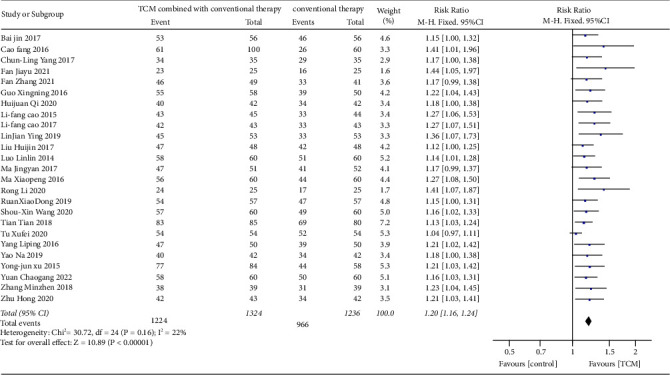
Effect of TCM combined with conventional therapy versus conventional therapy on the total effective rate.

**Figure 5:**
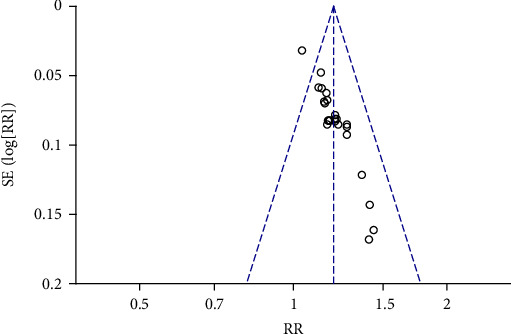
The funnel plot of TCM combined with conventional therapy versus conventional therapy on the total effective rate.

By a comparison of total effective rate, a total of 25 trials [19–30], [32–44] showed that TCM combined with conventional therapy was more effective than conventional therapy alone. There was no heterogeneity of the 25 trials by comparing total effective rate (Heterogeneity: Chi^2^ = 30.72, df = 24 (*P* = 0.16); I^2^ = 22%) with the fixed-effects model (RR = 1.20, CI 1.16 to 1.24; *p* < 0.001) used (Figure 4). The funnel plot showed that there might be a publication bias for the outcome of total effective rate (Figure 5). Subgroup analysis was performed by the commonly used Chinese medicine formulations to explore the possible source of heterogeneity. The results of subgroup analysis for Shenling Baizhu granule, Xingpi Yanger granule, Weichangan pill, and other Chinese medicine formulations were, respectively, RR = 1.17 (CI 1.08 to 1.27; *p* < 0.001, I^2^ = 22%, *P*_*He*_ = 0.83), RR = 1.19 (CI 1.12 to 1.27; *p* < 0.001, I^2^ = 3%, *P*_*He*_ = 0.40), RR = 1.20 (CI 1.09 to 1.31; *p* < 0.001, I^2^ = 0%, *P*_*He*_ = 0.94), and RR = 1.20 (CI 1.09 to 1.31; *p* < 0.001, I^2^ = 0%, *P*_*He*_ = 0.94). From the results mentioned above, we can conclude that the total effective rate of TCM in the treatment of AAD was significant, and the results are stable and reliable.

### 4.2. Effect of TCM Combined with Conventional Therapy on the Time to Change the Shape of Feces

**Figure 6:**
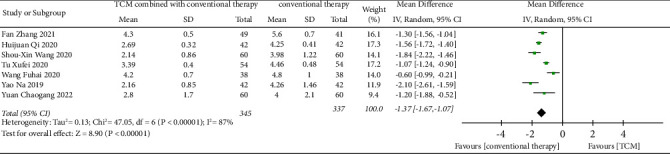
Effect of TCM combined with conventional therapy versus conventional therapy on the time to change the shape of feces.

By a comparison of the time to change the shape of feces, a total of 7 trials [23–25, 30–32, 34] showed that TCM combined with conventional therapy was more effective than conventional therapy alone. There was high heterogeneity of the 7 trials by comparing the time to change the shape of feces (Heterogeneity: Tau^2^ = 0.13; Chi^2^ = 47.05, df = 6 (*p* < 0.001); I^2^ = 87%) with the random-effects model (MD = −1.37, CI −1.67 to −1.07; *p* < 0.001) used (Figure 6). Due to the limited number of included studies, we did not detect the publication bias and did not conduct subgroup analysis.

### 4.3. Effect of TCM Combined with Conventional Therapy on Antidiarrheal Time

**Figure 7:**
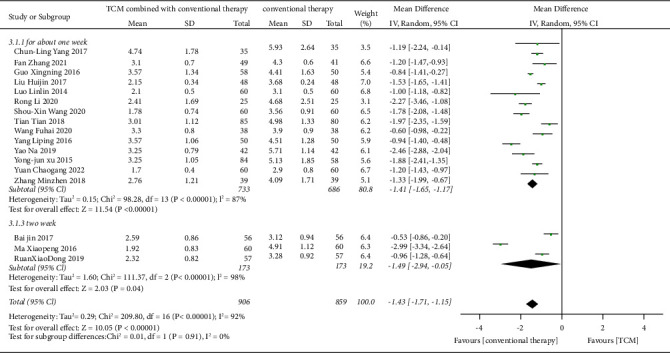
Effect of TCM combined with conventional therapy versus conventional therapy on antidiarrheal time.

**Figure 8:**
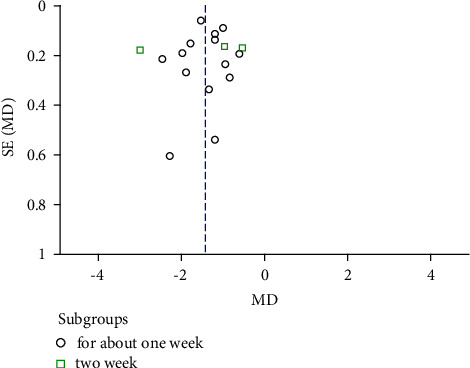
The funnel plot of TCM combined with conventional therapy versus conventional therapy on antidiarrheal time.

By a comparison of antidiarrheal time, a total of 17 trials [19, 20], [22–31], [33, 35, 37, 38, 40] showed that TCM combined with conventional therapy was more effective than conventional therapy alone. There was high heterogeneity of the 17 trials by comparing the antidiarrheal time (Heterogeneity: Tau^2^ = 0.29; Chi^2^ = 209.80, df = 16 (*p* < 0.001); I^2^ = 92%) with the random-effects model (MD = −1.43, CI −1.71 to −1.15; *p* < 0.001) used (Figure 7). The funnel plot showed that there was no publication bias for the outcome of the antidiarrheal time (Figure 8).

According to the commonly used Chinese medicine formulations and the intervention time, we conducted subgroup analysis. When the intervention time was two weeks, the result was RR = −1.49 (CI −2.94 to −0.05; *p* = 0.004, I^2^ = 98%, *P*_*He*_ < 0.001). The result was RR = −1.41 (CI −1.65 to −1.17; *p* < 0.001, I^2^ = 87%, *P*_*He*_ < 0.001). We performed a subgroup analysis by the time of intervention, but the heterogeneity was not reduced. Subgroup analysis by the commonly used Chinese medicine formulations showed that the results for Weichangan pill, Xingpi Yanger granule, and Shenling Baizhu granule were, respectively, RR = −0.68 (CI −0.96 to −0.40; *p* < 0.001, I^2^ = 0%, *P*_*He*_ = 0.416), RR = −1.96 (CI −2.20 to −1.72; *p* < 0.001, I^2^ = 95.7%, *P*_*He*_ < 0.001), and RR = −1.65 (CI −1.93 to −1.37; *p* < 0.001, I^2^ = 92.6%, *P*_*He*_ < 0.001). Therefore, subgroup analysis by the Chinese medicine formulations and the time of intervention hardly reduced the heterogeneity.

### 4.4. Effect of TCM Combined with Conventional Therapy on Other Indices

The pooled results showed that there were no significant difference between the two groups in CD3+, CD4+, CD8+, CD4 : CD8, time for bowel sounds to return to normal, hs-CRP, and IgM. There was significant difference between the two groups in frequency of diarrhea on the third day after TCM intervention, vomiting improvement time, diamine oxidase, IL-8, TNF, IgA, IgG, and average hospital stay. The details are shown in Table 3. There was no report about the adverse events.

## 5. Discussion

This systematic review was aimed at evaluating the effect of TCM combined with conventional therapy on treating AAD in children. Two systematic reviews have reported that TCM interventions combined with conventional therapy can be more effective than conventional therapy alone on treating AAD [45, 46]. However, there has been no systematic review and meta-analysis of TCM interventions treating AAD in children. In our systematic review of 26 RCTs involving 2636 children with AAD, it was demonstrated that TCM interventions combined with conventional therapy could effectively treat AAD in children.

With the overuse of antibiotics, a series of side effects are caused, such as antibiotic-associated diarrhea (AAD), which imposes more economic burden on the patient [47]. To our knowledge, the cause of AAD is commonly identified as intestinal flora disturbance [48]. As one of the oldest medical practices in human history, TCM has been widely used in diagnosing and treating diseases, which can improve the imbalance of intestinal flora [49, 50]. By conducting a systematic review, Zheng et al. [9] found that TCM can make an influence on modulating gut microbiota and improving glucose metabolisms in T2DM patients. Guo et al. [47] have demonstrated that Buzhongyiqi decoction (BD), Sijunzi decoction (SD), and Shenlingbaizhu decoction (SHD) can alleviate diarrhea symptoms induced by ceftriaxone sodium in animal experiments by balancing intestinal flora and improving villi structure. Hui et al. [48] reported that Qiweibaizhusan can restore and adjust the diversity of bacteria in mice intestine with diarrhea syndrome caused by administrating the mixture of gentamycin sulfate and cefradine. It is consistent with the results obtained by our systematic review that Shenling Baizhu granule, Xingpi Yanger granule, Erxieting granules, Huangqi Jianzhong decoction, Weichangan pill, Jianpi Bushen decoction, Modified Yigong powder, Xiaoer Fuxie powder, Guben Yichang tablets, Qiweibaizhu powder, and Buzhong Yiqi particles can significantly improve total effective rate and shorten the time to change the shape of feces, vomiting improvement time, time for bowel sounds to return to normal, and antidiarrheal time. Those outcomes above reflected the changes of AAD symptoms. The shortening of those outcomes could be associated with the reducing of average hospital stay and contribute to the enhancement of total effective rate in AAD. In addition, those herbal formulas are consisted of some typical herbs commonly used for tonifying Qi. Those are consistent with reports in previous studies that a major proportion of AAD is composed of the spleen deficiency type [51, 52]. All in all, the results have demonstrated that TCM could be used as an alternative therapeutic approach to treat AAD in children by balancing intestinal flora. However, the potential publication bias and heterogeneity may reduce the reliability of the results.

AAD is often accompanied by systemic inflammation, characterized by a significant increase in proinflammatory factors and a significant decrease in anti-inflammatory factors [53]. Changes in proinflammatory cytokines include tumor necrosis factor and interleukins, both of which play a primary role in communicating with immune cells and reflect inflammatory infiltration in the host [54]. Therefore, the levels of inflammatory cytokines are evaluated in AAD, including TNF and IL-8. In our results, TCM can improve some inflammation factors, such as TNF and IL-8, while TCM makes no difference in hs-CRP, which is consistent with the results reported by Ya-Nan et al. [55]. However, due to the limited study number, we cannot draw a definite conclusion about the results so far. In addition, due to the complexity of the chemical components in the formulas of herbs, it is difficult to make it clear about the mechanisms, restricting its clinical application at this stage.

The damage of intestinal mucosal barrier dysfunction can be also an important reason for AAD [47]. As one of the marker enzymes in many mammalian intestinal mucosal epithelial villus cells, DAO levels can objectively present the degree of intestinal mucosal damage [56]. Our results show that TCM can significantly reduce the level of diamine oxidase, which is the same with the results reported by Li et al. [57]. However, the small number of included studies limit the reliability of the results so far.

Intestinal immunity in the intestinal mucosa is the largest and most complex part of the overall immune system, accounting for at least 80% of all antibodies in adults [58]. As immune proteins are secreted by B lymphocytes when they differentiate into plasma cells, IgM, IgG, and IgA are important members of body humoral immunity, and their levels are positively correlated with body immunity [59]. As the body's immune system of the major groups of cells, T cell subgroup of CD4+ cells activate macrophages, CD8+ cells, by contrast, have inhibitory effect on cell activation [60]. AAD is closely linked to the disturbance of intestinal immunity [58]. In our systematic review, CD3+, CD4+, CD8+, CD4 : CD8, IgM, IgA, and IgG are summarized about the effect of TCM on AAD. However, the results in our systematic review indicate that TCM makes no significant difference on those outcomes. This may be associated with the small sample sizes in the included literature.

### 5.1. Limitations

In our systematic review, we focused on the effect of TCM on AAD in children. Nevertheless, the methodological quality scores were low and may restrict the generalizability of the results, which was the one of the main limitations in this systematic review. In addition, the studies were conducted in China, and the effect has not been established for other ethnic groups, which deserves further study. The publication bias and high heterogeneity may further reduce the reliability of the results. Therefore, more standard and large randomized controlled trials are needed in the future.

## 6. Conclusions

According to this systematic review, TCM interventions combined with conventional therapy can effectively alleviate AAD by improving diarrhea symptoms and reducing some inflammatory biomarkers in children. However, for the methodological quality is low which may reduce the reliability of the results, large and multicenter randomized controls are needed in the future.

## Figures and Tables

**Table 1:** Search terms.

#1	“traditional Chinese medicine” or “Chinese patent medicine”
#2	“Chinese herbal compound prescription” or “TCM” or “Chinese medicinal herb” or “Chinese herbal medicine”
#3	#1 or #2
#4	“Chinese”
#5	“formula” or “decoction” or “drug” or “prescription” or “medicine”
#6	#4 AND #5
#7	#3 or #6
#8	“controlled clinical trial” or “random∗” or “randomized controlled trial” or “RCT” or “trial”
#9	“antibiotic-associated diarrhea”
#10	#7 AND #8 AND #9

**Table 2:** Summary of included studies.

Author, year	Sample size		Interventions	Control	Treatment course	Outcome
	TCM	CG				
Zhu Hong, 2020	43	42	Shenling Baizhu granule + *a*	a	2 weeks	①④
Yao Na, 2019	42	42	Shenling Baizhu granule + *a*	a	5 days	①②⑤⑥
Wang Fuhai, 2020	38	38	Shenling Baizhu granule + *a*	a	5 days	②③④⑤⑦⑧
Luo Linlin, 2014	60	60	Shenling Baizhu granule + *a*	a	5 days	①②⑥
Shou-Xin Wang, 2020	60	60	Xingpi Yanger granule + *a*	a	7 days	①②⑤⑪
Tian Tian, 2018	85	80	Xingpi Yanger granule + *a*	a	5-7 days	①②③⑧⑫⑬
Huijuan Qi, 2020	42	42	Xingpi Yanger granule + *a*	a	5 days	①⑤⑨⑪⑫
LinJian Ying, 2019	53	53	Xingpi Yanger granule + *a*	a	3 days	①
Rong Li, 2020	25	25	Xingpi Yanger granule + *a*	a	3∼5 days	①②⑫⑬
Liu Huijin, 2017	48	48	Xingpi Yanger granule + *a*	a	7 days	①②⑧
Zhang Minzhen, 2018	39	39	Jianpi Bushen decoction + *a*	a	7 days	①②③
Fan Zhang, 2021	49	41	Erxieting granules + *a*	a	72 h	①②③⑤⑨
Cao fang, 2016	100	60	Erxieting granules + *a*	a	72 h	①
Yuan Chaogang, 2022	60	60	Huangqi Jianzhong decoction + *a*	a	6 days	①②⑤
Yang Liping, 2016	50	50	Weichangan pill + *a*	a	7 days	①②③⑮
Guo Xinning, 2016	58	50	Weichangan pill + *a*	a	7 days	①②
Ma Jingyan, 2017	51	52	Weichangan pill + *a*	a	7 days	①
Chun-Ling Yang, 2017	35	35	Jianpi Bushen decoction + *a*	a	72 h	①②
Yong-jun xu, 2015	84	58	Sijunzi decoction + *a*	a	5–10 days	①②
Tu Xufei, 2020	54	54	Modified Yigong powder + *a*	a	7 days	①⑤③
RuanXiaoDong, 2019	57	57	Xiaoer Fuxie powder + *a*	a	2 weeks	①②⑬⑨⑩
Bai jin, 2017	56	56	Xiaoer Fuxie powder + *a*	a	2 weeks	①②⑬⑨⑩
Li-fang cao, 2017	43	43	Yiersan + *a*	a	5 days	①
Ma Xiaopeng, 2016	60	60	Guben Yichang tablets + *a*	a	2 weeks	①②④⑦⑬⑨
Fan Jiayu, 2021	25	25	Buzhong Yiqi particles+ *a*	a	1 week	①
Li-fang cao, 2015	45	44	Qiweibaizhu powder+*a*	a	10 days	①

a: conventional therapy: antidiarrheal, fluid, diet adjustment, correct electrolyte imbalance, probiotics, montmorillonite powder, and so on; CG: control group. ①total effective rate; ②Antidiarrheal time; ③Diarrhea frequency; ④T cell subset: CD3+, CD4 +, CD8 +, CD4 +/CD8 +; ⑤The time to change the shape of feces; ⑥Time for bowel sounds to return to normal; ⑦Immune indice: CD3+, CD4+, IgA, IgG, IgM; ⑧intestinal barrier function: bacterial endotoxin(BT), diamine oxidase(DAO), D-Lactic acid(D-LC); ⑨Vomiting improvement time; ⑩Inflammatory factors: hs-CRP, IL8, IL-12, and TNF-*α*; ⑪Gastrointestinal hormones: gastrin(GAS), motilin(MOT), somatostatin(SS); ⑫Average hospital stay; ⑬Normal temperature time.

**Table 3:** Effect of TCM combined with conventional therapy on other indices.

Outcome or Subgroup	Studies	Participants	Effect Estimate(Mean Difference, 95% CI)	Heterogeneity
Frequency of diarrhea on the third day after TCM intervention	5	509	−1.18 [−1.77, −0.58]	96%
The change of CD3+	2	205	6.40 [−8.77, 21.57]	0%
Time for bowel sounds to return to normal	2	204	−6.65 [−17.27, 3.96]	97%
The change of CD4+	3	281	5.99 [−2.42, 14.41]	0%
The change of CD8+			−1.86 [−7.90, 4.18]	0%
The change of CD4 : CD8			0.19 [−0.12, 0.49]	0%
Effect of vomiting improvement time	3	346	−1.14 [−1.64, −0.64]	86%
The change of diamine oxidase	2	241	−3.69 [−6.45, −0.92]	0%
IgA	2	196	0.56 [0.02, 1.10]	0%
IgG	2	196	3.49 [0.69, 6.29]	0%
IgM	2	196	0.38 [−0.07, 0.83]	0%
Average hospital stay	3	299	−1.31 [−2.11, −0.51]	88%
Hs-CRP	2	226	−0.28 [−0.89, 0.34]	0%
IL-8			−1.16 [−2.07, −0.26]	0%
TNF			−0.83 [−1.12, −0.54]	0%

## Data Availability

The datasets used in the present review are available from the corresponding author on reasonable request.
